# Esophageal cancer cells resistant to T-DM1 display alterations in cell adhesion and the prostaglandin pathway

**DOI:** 10.18632/oncotarget.24975

**Published:** 2018-04-20

**Authors:** Juliette Sauveur, Eva-Laure Matera, Kamel Chettab, Philippe Valet, Jerome Guitton, Ariel Savina, Charles Dumontet

**Affiliations:** ^1^ Centre de Recherche en Cancérologie de Lyon, INSERM U1052, CNRS 5286 University of Lyon, Lyon, France; ^2^ Institut des Maladies Métaboliques et Cardiovasculaires, INSERM U1048, Université de Toulouse, UPS, Toulouse, France; ^3^ Hospices Civils de Lyon, Centre Hospitalier Lyon-Sud, Laboratoire de Biochimie-toxicologie, Pierre Bénite, France; ^4^ Université Lyon, ISPBL, Faculté de Pharmacie, Laboratoire de Toxicologie, Lyon, France; ^5^ Institut Roche, Paris, France; ^6^ Hospices Civils de Lyon, Pierre Bénite, France

**Keywords:** esophageal cancer, HER2, T-DM1, resistance, focal adhesions

## Abstract

Trastuzumab-emtansine (T-DM1) is an antibody-drug conjugate that specifically targets HER2 thanks to its antibody component trastuzumab. In spite of responses to this novel agent, acquired resistance to treatment remains a major obstacle. Prolonged *in vitro* exposure of the gastroesophageal junction cancer cell line OE-19 to T-DM1, in the absence or presence of ciclosporin A resulted in the selection of two resistant cell lines to T-DM1. T-DM1-resistant cells presented an increased expression of adhesion genes, altered spreading and higher sensitivity to anoikis than parental cells. A resistant cell line showed decreased adhesion strength, increased migration speed and increased sensitivity to RhoA inhibition. Genes involved in the prostaglandin pathway were deregulated in resistant models. Addition of prostaglandin E_2_ to T-DM1 partially restored its cytotoxic activity in resistant models. This work demonstrates that T-DM1-resistance may be associated with alterations of cell adhesion and the prostaglandin pathway, which might constitute novel therapeutic targets.

## INTRODUCTION

The human epidermal growth factor receptor 2 (HER2) belongs to the ErbB/HER receptor tyrosine kinase family that is necessary during normal development and plays a role in the oncogenesis of different cancers. HER2 is overexpressed in approximately 20% of breast cancers and is associated with poor outcome and high risk of recurrence [1, 2]. Gastric and esophageal cancer have a 5-year survival rate of less than 20% [3, 4]. HER2 is overexpressed in approximately 20% of gastric cancer and 33% of gastroesophageal junction (GEJ) cancers [5]. Trastuzumab was approved in 2010 for the treatment of patients with HER2-overexpressing metastatic gastric or GEJ adenocarcinomas who have not received prior treatment for metastatic disease.

Trastuzumab-emtansine (T-DM1) is an antibody-drug conjugate (ADC) that targets HER2 thanks to its antibody component trastuzumab, linked to DM1 via a thioether non-cleavable linker. T-DM1 conserves the mechanisms of action of trastuzumab [6], combined to the antimitotic activity of DM1. DM1 is a derivative of maytansine, which is a potent antimitotic agent that binds to tubulin at the same site as *Vinca* alkaloids [7]. Once the ADC binds to HER2, internalization and processing are necessary for the release of the active metabolites. The lysine-*N*^ε^-SMCC-DM1 is the only metabolite present in quantifiable amounts after lysosomal degradation of T-DM1 [8]. In patients with HER2-positive metastatic breast cancer T-DM1 was approved as a second line therapy in 2013. T-DM1 has also demonstrated efficacy against HER2 overexpressing uterine, bladder, lung and gastric cancers, both *in vitro* and *in vivo* [9–12]. The efficacy of T-DM1 is currently being evaluated in patients with HER2-positive gastric cancer. Since several patients treated with T-DM1 will eventually develop resistance to therapy it is important to determine mechanisms of resistance to this agent.

The efficacy of anti-cancer agents is often limited by acquired resistance to treatment. The increased expression and activity of the ABC transporters is responsible for decreasing the intracellular concentration of cytotoxic agents by enhancing drug efflux [13]. Resistance to maytansinoids and antibody-maytansinoid conjugates has been reported to be mediated by MDR1 [14, 15]. Resistance to tubulin binding agents can be due to alterations in tubulin isoforms or mutations and alterations in microtubule-associated factors [16]. In patients receiving trastuzumab, resistance can be associated with HER2 shedding leading to a cleaved active form of HER2 [17]. Moreover, the epitope recognized by trastuzumab can be masked by molecules such as MUC4 [18]. Additionally, HER2 inhibition can be overcome by an intrinsic activation of HER2 downstream pathways, for example by PI3KCA mutation or loss of PTEN activity, or a by-pass of HER2 blockade by activation of HER1/3 or IGF1R [19].

Resistance mechanisms to ADC have not yet been extensively studied as they are relatively novel agents, although resistance to T-DM1 has been observed in pre-clinical and clinical reports [20, 12, 21]. *in vitro*, resistance to ADCs may involve alterations of the surface or intracellular targets or to an abnormal endosomal/lysosomal pathway activity, leading to low intracellular concentrations of the cytotoxic agent. Decreases in the expression of the surface targets CD30 and HER2 have been reported in lymphoma cell lines resistant to brentuximab vedotin (BV) [22] and breast cancer cell lines resistant to an analogue of T-DM1, respectively [23].

To investigate resistance mechanisms, we selected T-DM1 *in vitro* resistant models using a GEJ cancer cell line continuously exposed to incrementally increased concentrations, in the presence or absence of ciclosporin A, an MDR1 inhibitor. The characterization of the resistant cell lines revealed various alterations including modified expression of genes involved in adhesion and the prostaglandin pathways.

## RESULTS

### Selection of *in vitro* T-DM1 resistant models

OE-19 cells resistant to T-DM1 were selected by continuous exposure to the antibody-drug conjugate (ADC) in the absence or presence of the MDR1 modulator ciclosporin A (CsA). CsA was added simultaneously with T-DM1 at a non-toxic dose of 1 μg/ml. The initial concentration of T-DM1 was 20% of the IC50 for the OE-19 cell line and was gradually increased when stable cell survival was obtained. The final T-DM1 concentration reached was 0.3 nM, which corresponds to 6 times the IC50 of the parental cell line in a 6-day cytotoxicity assay. We obtained two *in vitro* OE-19 resistant models to T-DM1: OE-19 TR in the absence of CsA and OE-19 TCR in the presence of CsA. Parental sensitive cells were designated as OE-19 S cells.

### Sensitivity phenotype of resistant cell lines

**Figure 1:**
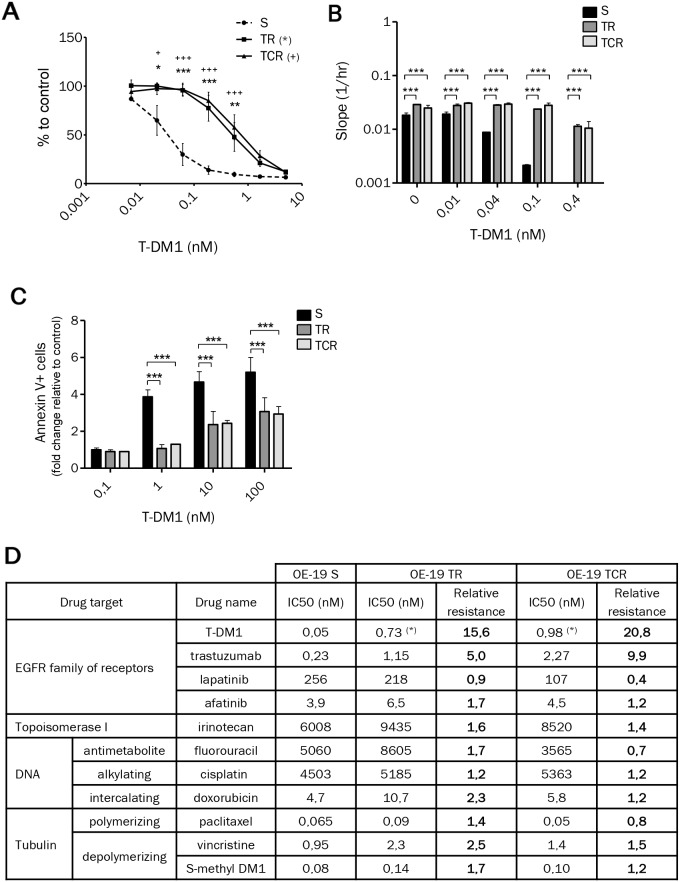
Chronic exposure to T-DM1 of OE-19 cell line results in resistance to this immunoconjugate **(A)** Cytotoxicity of T-DM1 on OE-19 S, TR and TCR cells determined by MTT cytotoxic assays revealed an increase in the IC50 of TR and TCR cells compared to parental cells. **(B)** Cytotoxicity of T-DM1 was studied using xCELLigence. The cell index slope was calculated using RTCA software and plotted. A single experiment is shown, representative of 3 experiments. The stronger the slope, the stronger the cell proliferation. **(C)** Cell death after 72h exposure to T-DM1 was assessed by annexin V staining using flow cytometry. The fold change in cell death relative to control was plotted for each cell line. The amount of cell death was decreased in TR and TCR compared to parental cells. Statistically significant differences were found for TR (^***^: P<0,001; ^**^: P<0,01; ^*^: P<0,05) and TCR (+) compared to S cells. **(D)** Parental and resistant OE-19 cells were exposed to the indicated anti-cancer agents and their sensitivity was assessed by MTT assay (or xCELLigence for trastuzumab). Data are shown as the mean IC50 calculated from 3-4 independent experiments and the relative resistance is the ratio of the IC50 for OE-19 TR or TCR over the IC50 for OE-19 S cell line (^*^: p<0,05).

We compared the sensitivity to T-DM1 of the selected resistant cells to that of sensitive parental cells using MTT cytotoxicity, xCELLigence and apoptosis assays. The IC50 of T-DM1 determined by the MTT assay was approximatively 16-fold higher in TR cells (0.73 nM) and 21-fold higher in TCR cells (0.98 nM) than in S cells (Figure 1A, Figure 1D). Real time monitoring by xCELLigence indicated that TR and TCR cells were capable of surviving under prolonged exposure to 0.1 nM T-DM1, unlike S cells (Figure 1B). Furthermore, apoptosis was quantified by annexin V staining after a 72h exposure to T-DM1 and we found that TR and TCR cell lines were less sensitive to T-DM1-induced apoptosis in comparison to S cells (Figure 1C). Using CFSE staining we verified that the changes observed where due to cell death and not to reduced proliferation (Supplementary Figure 1).

The sensitivity to HER2 targeted-therapy and standard chemotherapy of resistant cells was assessed by the MTT cytotoxicity assay and xCELLigence (Figure 1D). Cross-resistance to trastuzumab was observed in both T-DM1 resistant models, with an IC50 approximatively 5-fold higher in TR and 10-fold higher in TCR compared to OE-19 S cells. Both resistant models remained sensitive to DNA and tubulin targeting agents. These results suggest that T-DM1 resistant cells did not develop pleiotropic resistance mechanisms influencing cell death pathways and that the prolonged exposure to T-DM1 did not affect the sensitivity to other tubulin targeting or HER2 targeting agents.

### T-DM1 resistance is independent of drug efflux

**Figure 2:**
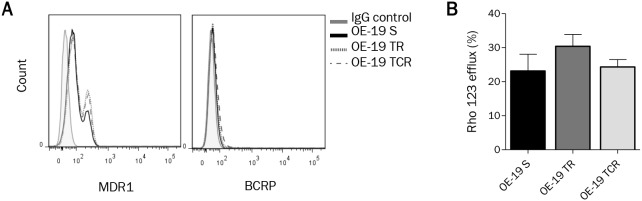
ABC transporters MDR1 and BCRP expression and activity are not significantly modified in resistance models **(A)** Surface expression of MDR1 and BCRP, studied by flow cytometry, does not show an increased expression of these ABC transporters in resistant cells. **(B)** Efflux activity was determined by Rhodamine 123 (Rho123) accumulation using flow cytometry. Rho123 efflux percent was not significantly different in resistant cells and in parental cells. The percentage of Rho123 efflux was calculated by comparing the mean fluorescence intensity (MFI) after uptakeand the MFI after efflux ((Uptake-Efflux)/Uptake^*^100).

The overexpression of ABC transporters is a well described mechanism conferring multidrug resistance. To examine whether resistance to T-DM1 was due to an increase in the expression and activity of efflux proteins, we studied two main ABC transporters Multidrug Resistance protein 1 (ABCB1, MDR1) and Breast Cancer Resistance Protein (ABCG2, BCRP). Using flow cytometry to detect the expression at the cell membrane, we found that MDR1 but not BCRP was expressed in parental and resistant cell lines (Figure 2A). Interestingly, we observed two distinct populations of cells which were MDR1^low^ and MDR1^high^ in OE-19 S. The amount of MDR1^high^ cells in TR and TCR cells was somewhat greater to that in S cells, suggesting that this population was slightly increased during selection of resistance models. To evaluate the activity of ABC transporters in parental and resistant cells, we performed a rhodamine 123 (rho 123) efflux assay (Figure 2B). Even though a subpopulation expressing MDR1^high^ was selected in TR and TCR cells, the efflux activity was not significantly increased in these resistant models. While these results do not suggest an increased drug-efflux activity in the resistant variants, we observed decreased accumulation of Lys-MCC-DM1 in the TCR cell line in comparison to the parental cell line (Supplementary Figure 2).

### Chronic exposure to T-DM1 does not affect HER2 expression or ability to bind antibody

**Figure 3:**
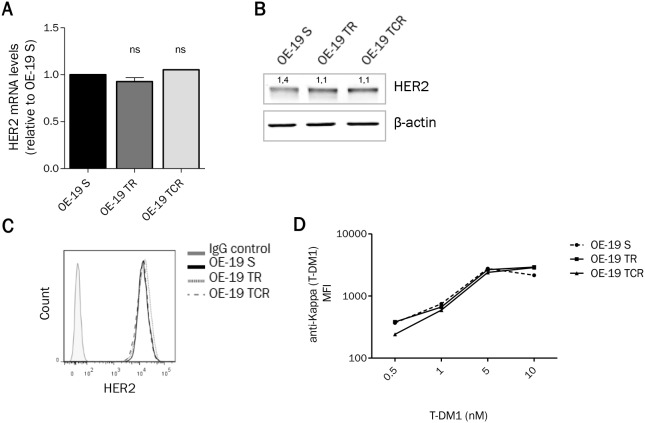
HER2 expression remains unchanged after chronic exposure to T-DM1 **(A)** mRNA and **(B)** proteinexpression from total cell lysates show that HER2 levels are unaffected in resistant cells. **(C)** HER2 expression at the cell surface determined by flow cytometry shows that parental and resistant cells express the same amount of HER2. **(D)** After exposure to T-DM1 for 1h at 4°C, cells were stained with anti-Kappa antibody and the mean fluorescence intensity (MFI) was studied using flow cytometry. T-DM1 was found to bind similarly parental and resistant cells. A single experiment is shown, representative of 3 experiments.

Since the antitumor activity of T-DM1 depends on its ability to bind to HER2, we studied the expression and accessibility of this target. The expression at the mRNA (Figure 3A) and protein (Figure 3B) levels, studied by immunoblotting and RT-qPCR respectively, was unchanged between parental and resistant cells. HER2 expression at the cell surface was studied by flow cytometry. No significant difference of HER2 surface levels was found between parental and resistant cell lines (Figure 3C), suggesting that resistance to T-DM1 was not due to decreased expression of HER2. However, the presence of HER2 at the cell surface does not infer that T-DM1 is able to bind to its target. We therefore studied T-DM1 binding by flow cytometry using an anti-kappa antibody and found that T-DM1 binds similarly to parental and resistant cells (Figure 3D). These results suggest that resistance to T-DM1 did not arise from downregulation or masking of HER2.

### T-DM1-induced cell cycle arrest is reduced in resistant models

**Figure 4:**
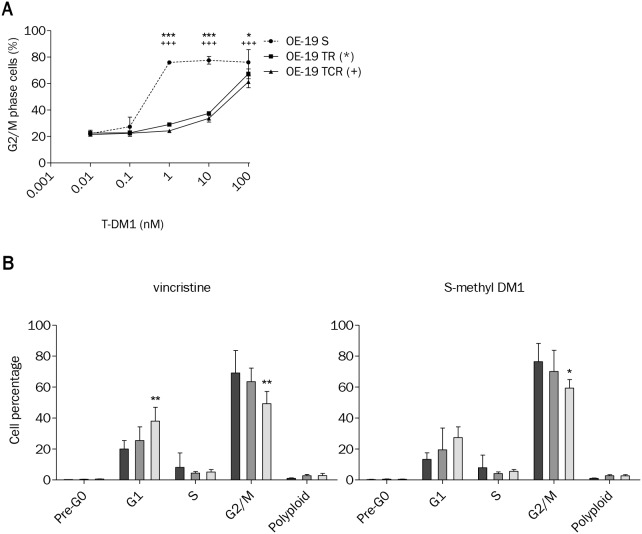
T-DM1-induced cell cycle arrest is impaired in OE-19 TR and TCR compared to OE-19 S **(A)** Exposure to increasing concentrations of T-DM1 for 24h and analysis of cell cycle distribution shows that the G2/M population was decreased in resistant cells compared to parental. Statistically differences are shown for TR (^***^: P<0,001; ^*^: P<0,05) and TCR (+++: P<0,001) compared to S. **(B)** Cell cycle distribution was studied by propidium iodide staining using flow cytometry after 24h exposure to 1 μM vincristine or 10 nM S-methyl DM1. Control conditions are not shown; only OE-19 S, TR and TCR exposed to drugs are plotted. Vincristine and S-methyl DM1 induced G2/M phase arrest in parental and resistant cells. The TCR cell line showed decreased sensitivity to cell cycle arrest in comparison to the parental cell line (^*^: P<0,05; ^**^: P<0,01).

We evaluated the effect of T-DM1 on cell cycle distribution by flow cytometry after propidium iodide staining. Parental cells were arrested in G2/M phase after 24h exposure to T-DM1, S-methyl DM1 and vincristine (Supplementary Figure 3). Cell cycle arrest was decreased in resistant cells compared to parental cells after exposure to increasing concentrations of T-DM1 for 24h (Figure 4A). Interestingly, resistant cells were sensitive to G2/M arrest induced by both S-methyl DM1 and vincristine, but the G2/M fraction in TCR was slightly inferior to that of the parental and TR cell lines (Figure4B). The absence of cell cycle arrest in the presence of T-DM1 may be due to a decreased concentration of the active metabolite or a difference in the microtubule dynamics of resistant cells compared to parental cells.

### Expression of βII and βIII isoforms, tubulin pools and post-translational modifications of tubulin are altered in T-DM1 resistant models

**Figure 5:**
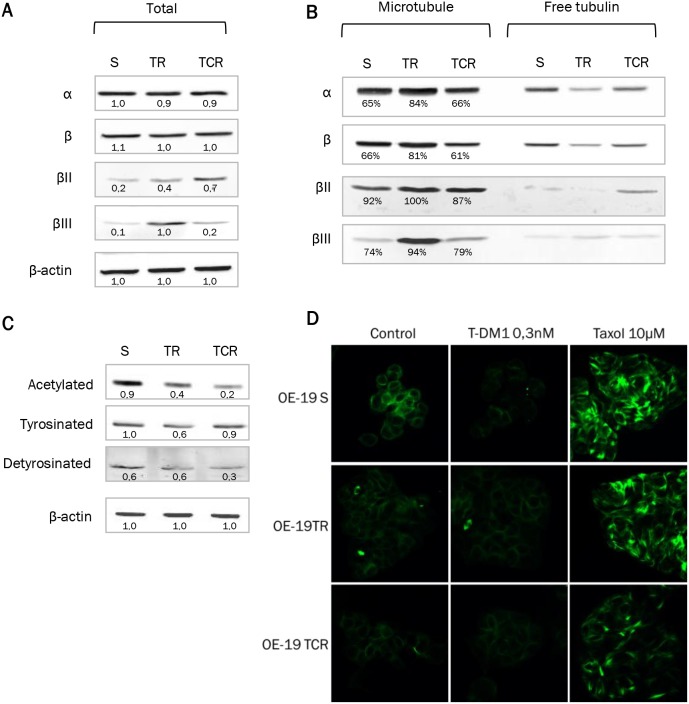
Tubulin expression and polymerized/soluble tubulin fractions in resistant models Expression of total α and β tubulin and isoforms βII and βIII was examined from total cell lysates or from purified fractions of tubulin. A single experiment is shown, representative of 3 experiments. **(A)** Protein levels from total cell lysate were studied by Western Blot and the density of the bands was normalized with actin to determine the expression fold-change. Cells resistant to T-DM1 express higher levels of βII and βIII tubulin than parental cells. **(B)** Protein expression after purification of polymerized (microtubule fraction) and soluble (free tubulin fraction) tubulin. The percentage of polymerized tubulin indicated on the Supplementary Figure hows that TR cells have an increased amount of polymerized tubulin compared to parental. **(C)** Acetylation, tyrosination and detyrosination state of α tubulin was studied by Western Blot. **(D)** Immunostaining of tubulin was performed in parental and resistant cells with or without exposure to T-DM1. Exposure to Taxol is shown as a positive control. Tubulin staining shows that the tubulin network is less sensitive to disruption by T-DM1 in resistant cells that sensitive cells.

Tubulin is the major intracellular target of T-DM1. Although prolonged exposure to T-DM1 did not affect total α and β tubulin protein content, isoforms βII and βIII were overexpressed in resistant models in comparison to parental cells (Figure 5A). Down-regulation of βIII tubulin by siRNA did not impact sensitivity to T-DM1 or T-DM1 induced cell cycle arrest in TR cells (Supplementary Figure 4). Hence, although βIII tubulin is increased in resistant models, its downregulation does not seem be sufficient to restore T-DM1-cytotoxicity.

To study tubulin pools, fractionation of polymerized tubulin (“microtubule”) and non-polymerized tubulin (“free tubulin”) was performed (Figure 5B). We found that α and β tubulins were increased in the microtubule fraction in TR cells but remained unchanged in TCR cells, compared to S cells. Approximately 80% of tubulin was polymerized in TR cells versus 65% in parental and TCR cell lines. This was associated with a decrease in the free tubulin fraction, confirming that a larger proportion of tubulin was present under polymerized form in the TR resistant cells. Isoforms βII and βIII were predominantly present in microtubules in both parental and resistant cell lines. As post-translational modifications (PTMs) of tubulin may affect microtubule dynamics, we studied the acetylation and tyrosination status of α- tubulin by Western Blot. We found a decreased amount of acetylated tubulin in TCR cells and detyrosinated tubulin in both resistant models (Figure 5C). Also, the tubulin network was less sensitive to disruption by T-DM1 in resistant cells that sensitive cells as shown by tubulin staining (Figure 5D). Hence, the modifications of PTMs in resistant cells are likely to be related to altered microtubule dynamics in these cell lines.

### Deregulation of adhesion genes is associated with alterations in cell morphology and migration, and shape and strength of focal adhesions

**Figure 6:**
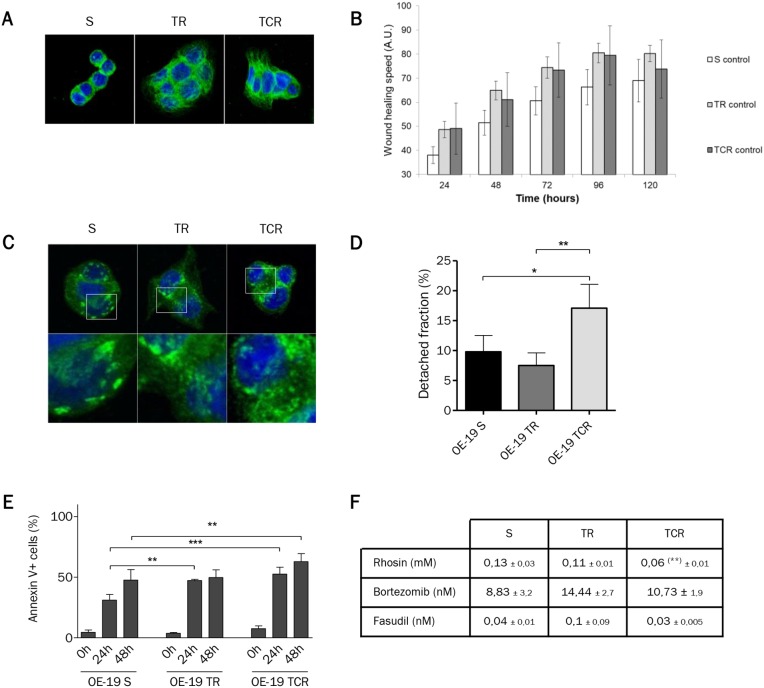
Morphology is modified in both resistant cell lines but only TCR cells have increased migration speed and decreased adhesion strength **(A)** Immunofluorescence staining of α-tubulin (green) and DAPI (blue) observed by confocal microscopy shows morphological differences between parental and T-DM1-resistant cell lines. **(B)** Migration speed determined by wound healing assay shows an increase in TR and TCR cells compared to parental **(C)** Immunofluorescence staining of talin (green) and DAPI (blue) observed using a confocal microscope. Focal adhesions in parental cells appear bigger and in least amount that those in resistant cells. **(D)** Detached fraction of parental and resistant cells following detachment by centrifugal force shows a decreased adhesion strength in TCR cells (^*^: P<0,05; P<0,01). **(E)** Cell death was quantified by Annexin/PI staining following 24h and 48h of incubation in suspension conditions. The percent of living cells was decreased in TR cells at 24h (P<0,01) and in TCR cells after 24h (P<0,001) and 48h (P<0,01) compared to parental. **(F)** Sensitivity to rhosin, bortezomib and fasudil was studied using MTT cytotoxic assays after 6 days exposure to the corresponding cytotoxic agents. Sensitivty to rhosin was increased in TCR cells compared to parental (^**^:P<0,01).

To gain insight into the resistance mechanisms in TR and TCR cell lines, we performed a pangenomic transcriptomic analysis of OE-19 S, TR and TCR. Bioinformatic analysis OE-19 TR and TCR versus OE-19 S allowed the identification of numerous genes involved in adherens junctions, ECM-receptor interaction, cell adhesion molecules and focal adhesion (Table S1A). To validate these results, we studied the differential expression in resistant cells by RT-qPCR of tyrosine kinase receptors (*EGFR* and *MET*), actin-interacting molecules (*ACTN1* and *VCL*), and regulators of actin cytoskeleton (*ROCK1*, *RAC2* and *DIAPH1*). We found that *EGFR*, *MET*, *ROCK1*, *DIAPH1*, *ACTN1* and *VCL* were upregulated while *RAC2* is downregulated in resistant cells (Table S1B). We examined cell morphology by immunofluorescent staining of α- tubulin and found that parental cells are spread in a round shape while both resistant cell lines spread in a polygonal shape (Figure 6A, Supplementary Figure 7A). To confirm that the differences in morphology were due to a difference in adhesion, we verified that cell size remained unchanged by mean diameter of suspension cells (Supplementary Figure 5). Next, we assessed the migration capacity by wound-healing assay and we found that although parental cells were capable of migrating, TR and TCR cells closed the wound faster (Figure 6B). We verified that the changes observed in the wound healing experiment where not due to a different proliferation rate of resistant cells lines by CFSE staining (Supplementary Figure 1). We studied the shape of focal adhesions by immunostaining of talin (Figure 6C, Supplementary Figure 7B) and found that while the total expression of talin appeared to be greater in the resistant cells (Supplementary Figure 8), the size of focal adhesions seems to be reduced in resistant cell lines, in particular in TCR cells. Also, it appeared that the number of focal adhesions was increased in TR and TCR cells. Finally, we measured the adhesion strength using a centrifugal-force based adhesion assay (Figure 6D). Following overnight adhesion, the detached fraction was increased in TCR cells and unchanged in TR cells compared to parental. Although spreading, migration speed and focal adhesions were modified in both resistant cell lines, only TCR cells showed decreased adhesion strength.

To evaluate the implications of focal adhesion changes in resistant cells, we assessed their viability under suspension conditions. Cell death was studied after plating cells in low-adherent condition plates for 24h and 48h. We found fewer living cells in both resistant cell lines compared to parental cells (Figure 6E). This result suggests that resistant cells to T-DM1 are more dependent upon adhesion for survival. Then, in order to determine the relationship between adhesion and resistance we studied the sensitivity of cells to different inhibitors of focal adhesion points. We inhibited RhoA, a major regulator of the actin cytoskeleton, using rhosin and two of its targets, ROCK1 by fasudil and FAK by bortezomib. We found that TCR showed slightly increased sensitivity to rhosin in comparison to parental cells (Figure 6F). However, the sensitivity to ROCK1 or FAK inhibitors was unchanged in resistant cells compared to parental cells.

### Prostaglandin E_2_ increases sensitivity to T-DM1 of OE-19 resistant cell models

**Figure 7:**
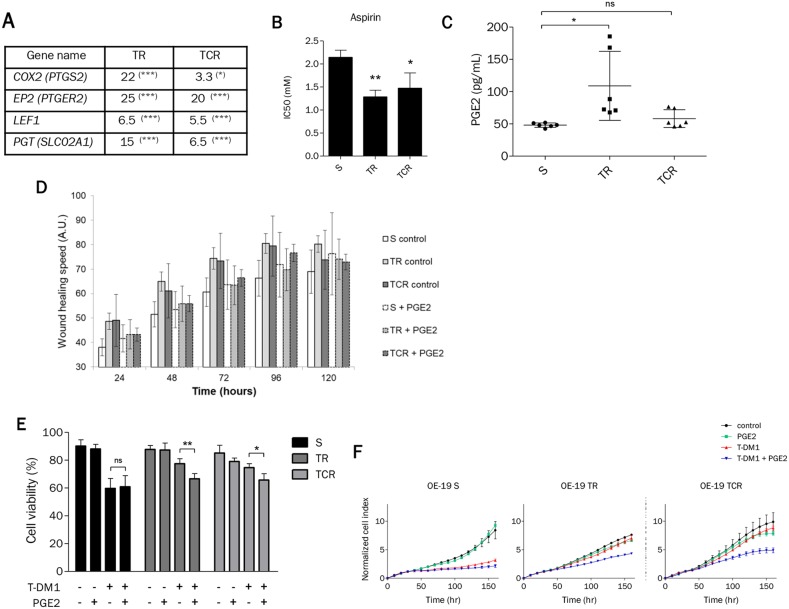
Prostaglandin E_2_ mediates sensitivity to T-DM1 in resistant cells **(A)** The expression of genes involved in the prostaglandin pathway is highly modified in resistant cells to T-DM1. The results shown are the expression fold changes of TR or TCR over parental cells for the indicated genes determined by RT-qPCR. **(B)** Sensitivity to aspirin studied by MTT assay was found to be increased in TR and TCR cells compared to S cells (^*^: P<0,05; ^**^: P<0,01). **(C)** Quantification by ELISA of PGE2 in the supernatant of each cell line shows an increased amount in TR cells compared to parental cells. **(D)** Wound healing assay performed on OE-19 S, TR and TCR cell lines in the absence or presence of 10 μM PGE_2_. The addition of PGE_2_ decreased the migration speed of the parental cell line but had no effect on the migration speed of both resistant cell lines. **(E)** Cell survival after exposure to 1 nM T-DM1 and 10 μM PGE_2_ was studied by Annexin V/PI staining after 72h exposure. The presence of PGE_2_ increases the sensitivity to T-DM1 of resistant models. **(F)** The increased sensitivity to T-DM1 in the presence of PGE_2_ was confirmed by xCELLigence. After overnight incubation cells were exposed to 1 μM PGE_2_ and/or 0,1 nM T-DM1. The normalized cell index of TR and TCR cells exposed to T-DM1 and PGE_2_ is inferior to control, PGE_2_ and T-DM1.A single experiment is shown, representative of 3 experiments.

The transcriptomic analysis revealed that the prostaglandin pathway was deregulated in cells resistant to T-DM1. The overexpression of *COX2*, *EP2, LEF1* and *PGT* was confirmed by RTqPCR (Figure 7A). Prostaglandins (PGs) are synthetized from arachidonic acid by cyclooxygenase enzymes (COX1 and COX2), and we found that *COX2* gene expression was increased by 22-fold change in TR (P value=0.0005) cells and 3.3-fold change in TCR cells (P value=0.0134). *COX2* expression is inducible and the promoter region contains a TCF/LEF response element. Since *LEF1* is overexpressed in resistant models, it could be involved in the increased expression of *COX2*. The *PGT* or *SLC02A1* gene coding for an SLC transporter that mediates the energy-dependent export of prostaglandins [24], was transcriptionally increased 15-fold in TR (P value=0.0002) and 6.5-fold in TCR cells (P value<0.0001). The *EP2* gene coding for prostaglandin E_2_ receptor, was transcriptionally increased 25-fold in TR cells (P value=0.0002) and 20-fold in TCR cells (P value<0.0001). Since *COX2* was over-expressed in cells resistant to T-DM1, we studied their sensitivity to aspirin, an inhibitor of COX1 and COX2 that blocks the synthesis of all PGs. We found a small yet significant decrease in the IC50 values of resistant cell lines compared to parental (Figure 7B).

In view of these results, we quantified the extracellular PGE_2_ for each cell line and found that the amount of PGE_2_ was increased in the TR cell supernatant in comparison to that of S cells (Figure 7C). Interestingly, the increased amount of PGE_2_ in TR cells is associated with higher *COX2* and *PGT* expression than in TCR and S cells. Since PGs are involved in cell adhesion, spreading and migration, we exposed parental and resistant cell lines to PGE_2_ to study their migration in a wound healing assay (Figure 7D). Results suggest that the addition of PGE_2_ delayed migration of both resistant cell lines but not the parental cell line. To assess whether PGE_2_ was involved in the resistance to T-DM1, we studied the sensitivity to T-DM1 in parental and resistant cells in the presence of PGE_2_ by Annexin/PI staining (Figure 7E) and xCELLigence assays (Figure 7F). We found that the presence of pharmacological concentrations of PGE_2_ increased the sensitivity to T-DM1 in resistant cells but not in parental cells. Additionally, exposure to PGE_2_ was associated with enhanced expression of PTGS2 in resistant cells but not in sensitive cells while SLCO2A1 was decreased specifically in OE19-TCR cells after combined exposure to PgE2 and T-DM1 (Supplementary Figure 9). Altogether these results indicate that the prostaglandin pathway, in particular the inhibition of the cyclooxygenases, may be an alternative target in T-DM1 resistant cells.

## DISCUSSION

The efficacy of cancer therapies is very often limited by acquired resistance. Although the exact mechanisms of resistance to T-DM1 have not been described, T-DM1 has often been found to lose benefit despite continued treatment in some patients [25, 26]. To better understand possible mechanisms of resistance to T-DM1, we performed *in vitro* selection of OE-19 cell line resistant to T-DM1 by prolonged exposure at low concentrations over several months in the absence or presence of CsA. CsA was added during selection of resistant cells to prevent MDR1-mediated resistance. However, neither TR nor TCR cell lines showed increased MDR1 expression or efflux activity, independently of the presence of CsA.

Reduced expression of the ADC target has previously been observed in some *in vitro* resistant models [22, 27]. Also, HER2 shedding leads to the expression of a cleaved transmembrane p95HER2 protein and a circulating extracellular domain and is responsible for the inefficacy of trastuzumab and possibly T-DM1 in patients [17, 18]. In our resistant models, HER2 expression remained unchanged as well as the ability of cells to bind T-DM1. Our results show that resistance to T-DM1 in TR and TCR cells did not arise from downregulation or masking of HER2 at the cell surface. Additionally, resistant cells retained sensitivity to lapatinib, a small molecule inhibitor of Her2, suggesting that the Her2 pathway remained functionally active.

Resistance to T-DM1 could be due to the inability of DM1 to bind to microtubules or reduced inhibition of microtubule dynamics by DM1. Total α and β tubulin content remained unchanged in resistance models, but the percentage of polymerized tubulin was increased in TR cells. As maytansine binds exclusively to soluble tubulin dimers it is possible that the reduction of the free tubulin pool in TR cells contributes to their resistance to T-DM1. Moreover, we found decreased acetylation of α-tubulin in both resistant cell lines, in particular in TCR cells. Tyrosination was slightly increased and detyrosination was decreased in TCR cells compared to parental cells. α-tubulin acetylation is associated with stable, long lived microtubules while newly assembled microtubules are highly tyrosinated [28, 29]. Hence, even though tubulin pools remained unchanged in TCR cells compared to S cells, PTMs indicate that microtubules in these cells seem to be less stable that in parental and TR cells. The contents of tubulin isoforms βII and βIII were increased in resistant cell types. βIII has been reported to possess specific characteristics in terms of microtubule dynamics and has also been reported to be associated with drug resistance both *in vitro* and *in vivo* [30–35]. However, the downregulation of βIII tubulin by siRNA in the TR cell line did not reverse the resistance to T-DM1 nor restored T-DM1-induced cell cycle arrest. This suggested that overexpression was not sufficient for resistance to T-DM1. Microtubules are constantly undergoing cycles of polymerization and depolymerization, called “dynamic instability” which are crucial to many of their functions, in particular chromosomal segregation during anaphase. Specific tubulin isoforms and tubulin PTMs have been reported to be associated with tubulin dynamics and possibly drug binding [36–38]. Overall, resistant cells displayed a number of microtubule-associated alterations but whether these play a role in the resistance phenotype or are consequences of exposure to T-DM1 remains to be determined.

Transcriptomic analysis of TR and TCR cells showed a deregulation of genes coding for adhesion molecules such as integrins and several regulators of the actin cytoskeleton (Supplementary Table 1A, Supplementary Figure 6). Also, we found that T-DM1 resistant cells have a different shape and increased migration speed compared to parental cells. McGrail et al. found that taxol resistance was associated with decreased adhesion strength and that cells presented small nascent adhesions, characterized by strong traction forces [39]. We found that TCR cells have decreased adhesion strength that coincided with smaller focal adhesions. These results suggest that TCR cells present nascent adhesions with strong traction forces, which matches with their increased migration speed. Besides cell motility, adhesion to the extracellular matrix (ECM) is necessary for survival. Cells that detach from the ECM rapidly undergo apoptosis [40], a phenomenon designated by Frisch and Francis as *anoikis* [41]. We found that resistant cells were more sensitive to *anoikis* than parental, which suggests that their survival may be dependent on signaling pathways triggered by adhesion molecules. We studied the response of TR and TCR cells to inhibitors of cell adhesion such as rhosin, an inhibitor of RhoA that blocks actin stress fiber formation and focal adhesion assembly [42] and we observed an increased sensitivity in TCR cells compared to parental cells. To identify RhoA downstream pathways responsible for TCR cell death, we inhibited two targets of RhoA. However, the inhibition of ROCK1 or FAK did not modify TCR cell death compared to parental cells, suggesting that the altered pathway involves other RhoA interactors. In depth studies need to be performed to validate the implication of cell adhesion genes in resistance to T-DM1.

Genes involved in the prostaglandins (PGs) pathway were upregulated in models resistant to T-DM1. PGs are bioactive lipids implicated in normal development and pathological processes such as inflammation and cancer [43]. The expression of COX2 is upregulated in many types of cancer and has been associated with decreased survival [43–45]. The upregulation of *COX2* in both resistant cell lines coincided with increased sensitivity to inhibition of cyclooxygenases by aspirin. Moreover, PGE_2_ has been described to be the most abundant PG in tumors [43], and its amount was increased in the supernatant of TR cells compared to parental cells. In view of this result and that the gene coding for its receptor (*EP2*) was upregulated in both resistant cell types, we studied its effect on T-DM1 resistant cells. We found that the addition of pharmacological concentrations of PGE_2_ increased the cytotoxic effect of T-DM1 while PGE_2_ alone had no impact on cell survival. PGE_2_ has been extensively studied in rodent experiments and in the clinic and shown to be involved in tumor growth and associated with poor prognosis [46, 47]. However, a dual role of PGE_2_ has been observed in some cases and reviewed by Greenhought et al [48]. Thus, PGE_2_ could have pro or anti-tumoral activities depending on the cell type and the experimental settings. PGE_2_ activates many downstream targets such as EGFR, MAPK, angiogenic and antiapoptotic factors, and chemokines [46]. These pathways could be implicated in resistance to T-DM1 and their modulation could restore sensitivity to T-DM1. Additional studies are required to describe the pathways involved in the role of PGE_2_ in this setting.

In summary our results show for the first time that resistance to an antibody-drug conjugate may be associated with modifications in cell adhesion and morphology as well as with alterations of the prostaglandin pathways. Future studies will help to determine the clinical relevance of these observations and whether it is possible to exploit these alterations as potential novel therapeutic targets.

## MATERIALS AND METHODS

### Cell culture

OE-19 cell line was purchased from ECACC, tested for *Mycoplasma* once a month and cultured in complete RPMI 1640 medium supplemented with 10% fetal calf serum and 100 μg/ml streptomycin. Counting was performed using Cellometer Auto T4 (Nexcelom Bioscience LLC). Cells were maintained at 37°C and 5% CO2 at all times. OE-19 cell line was exposed to increasing concentrations of T-DM1 for 6 months in the absence or presence of 1 μg/ml ciclosporin A (C3662; Sigma-Aldrich) to obtain resistance models.

### Chemotherapy and targeted agents

T-DM1 was kindly provided by Genentech and S-methyl DM1 by ImmunoGen.

### Cytotoxicity assay

Cell suspensions (100 μL) were inoculated in 96-well plates at a density of 2,500 cells per well and incubated overnight before exposure to therapeutic agents. After 6 days, cell viability was determined by the MTT (3-(4,5-dimethylthiazol-2-yl)-2,5-diphenyl tetrazolium bromide) assay, 20 μL of MTT solution were added to each well and cells were incubated at 37°C for 4h. Then, media/MTT mixture was removed and 100 μL of 4% HCl 1N/isopropanol were added to dissolve the purple formazan crystals. The absorbance was measured at 570 nm with 690 nm as a reference readout using a Thermo MultiSkan EX microplate reader. Percentage of living cells was calculated using the absorbance in drug-exposed cells over control cells. IC50 values were calculated using CompuSyn software.

### Real-time cell analysis (RTCA)

The xCELLigence RTCA DP instrument (ACEA Bioscience) was used to monitor cell impedance in real time. Cells were seeded (10,000 cells/well) in E-plate 16 and allowed to adhere overnight before adding the cytotoxic agents or PGE_2_. Cells were monitored for one week. The xCELLigence software calculates the slope of the impedance trace between two-time points (from exposure to T-DM1 to the end of experiment in our design)

### Efflux assay

Cell suspension was prepared at 4^e6^ cells/ml in RPMI media containing 0.5 μg/ml Rhodamine 123 (Santa Cruz, sc-208306) in the absence or presence of 3 μg/mL of CsA and incubated for 30 min at 37°C, 5% CO2. Cells were washed with cold RPMI media and 2^e5^ cells were suspended in RPMI media with or without 3 μg/mL of CsA and incubated for 90 min at 37°C, 5% CO2. Remaining cells were kept at 4°C. All conditions were washed twice with cold DPBS and analyzed by flow cytometry.

### Flow cytometry

Cells were incubated for 30 min at room temperature with the corresponding antibodies: HER2 (4225666), BCRP1 (561180) and anti-kappa (214561) from BD Bioscience, MDR1 (348608) from Biolegend and mouse IgG1 κ control isotypes from BD Pharmingen. CFSE staining was performed using Cell trace CFSE proliferation kit according to the manufacturer protocol (Invitrogen C34554). For apoptosis measurements, cells (2^e5^) were seeded in 6 well plates and incubated overnight. Then, T-DM1 was added to each well at increasing concentrations up to 100 nM for 72h. After incubation, cells were harvested, washed with cold DPBS + 10% SVF and stained using Annexin-V-FLUOS Staining Kit (Roche) according to the prescribed protocol and analyzed by flow cytometry. Annexin V positive cell percentages in conditions of exposure to T-DM1 were normalized to that of control for each cell line. For cell cycle distribution, cells (2^e5^) were seeded in 6 well plates and incubated overnight. Then, they were exposed to increasing concentrations up to 100 nM of T-DM1, 1 μM vincristine and 10 nM S-methyl DM1 for 24h. Cells were collected and incubated for 30 min at 4°C with propidium iodide (0.05 mg/mL) containing Nonidet-P40 (0.05%) and 4 μM of trisodium citrate. Cells were filtered using Falcon tubes with cell-strainer cap (352235) and analyzed by flow cytometry. Analyses were performed using a BD LSRII flow cytometer with BD FACSDiva software (BD Biosciences, San Diego, CA, USA) and FlowJo software (Tree Star, Ashland, OR, USA).

### Western blot

Proteins were extracted with RIPA buffer (RIPA buffer, 1 mM DTT, 1M NaF, 100 mM sodium orthovanadate and protease and phosphatase inhibitors). After SDS PAGE separation, and transfer onto a PVDF membrane by iBlot dry blotting system (Invitrogen), membranes were incubated overnight at 4°C with primary antibodies: HER2 (GTX50425; Genetex), βIII-tubulin (clone TUJ1), βII-tubulin (clone 7B9) α-tubulin (T6199), β-tubulin (T4026) and β-actin (A5441) from Sigma-Aldrich, and 1h at room temperature with secondary antibodies (IRDye Infrared Dyes from LI-COR Biosciences). Membranes were scanned using Odyssey infrared imaging system (LI-COR Biosciences) and densitometric quantification was performed with Odyssey software. Quantification of Western Blot was performed using Image J software (Rasband, W.S., National. Institutes of Health). Expression levels of proteins were normalized against β-actin.

### Separation of soluble tubulin and microtubules

Separation was performed as previously described [49]. Cells (20^e6^) were lysed in 300 μL of PEM 50DP Buffer (50mM Pipes, 1mM EGTA, 1mM MgSO4, 0.05% sodium azide, 1mM DTT and proteinase inhibitors at pH 6.7) by three freeze-thaw cycles. Cells were ultracentrifuged (100000 g for 1h at 20°C) and the supernatant was separated from the pellet. The pellet fraction was resuspended in 100 μL of PEM 50DP buffer, incubated on ice for 30 min for depolymerization and ultracentrifuged at 50000g for 45 min at 4°C to recover the supernatant containing “soluble tubulin”. The supernatant was incubated at 35°C with 1mM GTP for 30 min for polymerization and ultracentrifuged at 50000g for 45 min at 35°C. The supernatant was discarded and the pellet containing the “microtubules” was resuspended in 50 μL of PEM 50DP buffer. Alpha and beta tubulin isotypes were then analysed by Western Blot.

### Microarray

The microarray was performed by ProfileXpert-LCMT platform using OE-19 S, TR and TCR cell lines as previously described [50]. Data was analyzed with GeneSpring and Ingenuity softwares (Agilent Technologies, Santa Clara, CA, USA).

### RT-qPCR

RNA was extracted using the QIAamp RNeasy Mini Kit (QIAGEN). Random primers (Life Technologies) were used for reverse transcription. Primer sequences were based on Roche database and quantitative PCR was performed using the LightCycler 480 Real-Time PCR system (Roche Life Science, Indianapolis, IN, USA).

### Immunofluorescence

Immunostaining was performed as previously described [49]. The primary antibodies used were obtained from Sigma: the anti-α-tubulin (T6199 clone DM1A) diluted at 1/50 and anti-talin (T3287, clone 8d4) diluted at 1/100.

### Adhesion strength assay

The centrifugal force-based adhesion assay was based on previous published methods [39]. Briefly, cells were seeded at 20,000 cells/well in 96-well plate and left to adhere overnight in media without red phenol. Cells were stained with 2μM Calcein AM (Sigma-Aldrich, C1359) in PBS-dextrose 2mM for 20 min at 37°C and rinsed with PBS. Cells were covered with PBS-dextrose before an initial fluorescence reading at 485nm excitation, 535nm emission on a Plate Chameleon multilabel detection platform. Next, the supernatant was discarded, and the inverted plates were centrifuged at 60g for 5 min and rinse with PBS. Then, PBS-dextrose was added before a final reading. The detached fraction was calculated as 1-final fluorescence/ initial fluorescence.

### Wound healing assay

#### Scratch wound healing assay was performed using Incucyte, ZOOM System (Essen Bioscience), according to the manufacturer's protocol

#### PGE_2_ quantification

Cells were washed and suspended in SVF free media to plate 2^e5^ cells per well in a 12-well plate. Supernatants were recovered 24h after plating and PGE2 was quantified with ELISA High sensitivity (ENZO Life Sciences).

#### Quantification of Lys-MCC-DM1

Analysis was performed with a Q-Exactive-Plus (hybrid quadrupole-Orbitrap mass spectrometer) coupled with liquid chromatography (Ultimate 3000) from Thermo ScientificTM. Lys-MCC-DM1 was separated on a Hypercarb (5.0 μm, 150 mm x 2.1 mm i.d.) Thermo ScientificTM column. Gradient elution with water containing 0.1% formic acid (A) and acetonitrile containing 0.1% formic acid (B) was applied. The mobile phase was delivered through the column (temperature at + 30° C) at a flow rate of 250 μl/min. At start, (B) was maintained for 1.5 min at 30 % (v/v). After, (B) increased linearly until 3 min to 80 % (v/v) and this composition is fixed for 4 min. Then (B) was reset to 30 % (v/v) for 5 min. Analysis of Lys-MCC-DM1 was carried out in positive ion mode using a heated electrospray ion source. The signal of Lys-MCC-DM1 (C53H75N6O15ClS] was collected using target-sim mode with a resolution of 70,000 and following the [M+H]+ ion at m/z 1103.4765. Lys-MCC-DM1 eluted as sharp peak at 7.2 min and cell components did not interfere with the analysis. Cells were exposed to 5nM T-DM1 for one hour and washed twice with phosphate buffered saline and extracted with 300 μl of a mixture containing acetonitrile and water (80/20; v/v). The extract was transferred in Eppendorf tube, then shacked for 5 min, and conserved at −80°C until analysis. The day of analysis, samples were vigorously vortexed and centrifuged for 10 minutes at 13000 g. The supernatant was evaporated to dryness under nitrogen at 37°C. Finally, the residue was resuspended in 150 μL of water and 20 μL were injected into liquid chromatography-mass spectrometry device. For calibration curves, blank cell samples were spiked with the appropriately diluted standard solutions to final concentrations of 1.25, 2.5, 5, 12.5 and 25 ng/ml. Calibration curves were constructed by plotting the ion abundance peak area as function of cell Lys-MCC-DM1 concentration. Data were fitted by weighted (1/concentration) for least-squares regression, and standard curves were determined using linear regression analysis.

### Statistical analysis

Experiments were performed at least three times and shown in graphs as the mean ± SD. Graphs and statistics were done using GraphPad Prism software. Statistics on cell survival experiments such as AnnexinV/PI staining or MTT assay were calculated by Two Way Anova followed by Bonferroni post-test. Statistics on gene expression by RT-qPCR were performed by Student t test.

## SUPPLEMENTARY MATERIALS FIGURES AND TABLES





## Abbreviations

ADC: (Antibody-drug conjugate)
BV: (Brentuximab vedotin)
ECM: (Extra-cellular matrix)
GEJ: (Gastroesophageal junction)
HER2: (Human Epidermal growth factor Receptor 2)
PGs: (Prostaglandins)
PGE2: (Prostaglandin E2)
PTMs: (Post-translational modifications)
T-DM1: (Trastuzumab emtansine)
