# Supplementary material for: Esophageal cancer cells resistant to T-DM1 display alterations in cell adhesion and the prostaglandin pathway
Source: Oncotarget. 2018 Apr 20;9(30):21141–55. doi: 10.18632/oncotarget.24975 (PMC5940380; doi:10.18632/oncotarget.24975)
Supplement: Supplementary file 1 [file oncotarget-09-21141-s001.pdf]

# Esophageal cancer cells resistant to T-DM1 display alterations in cell adhesion and the prostaglandin pathway

## SUPPLEMENTARY MATERIALS

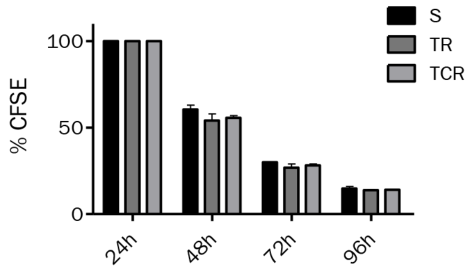


**Supplementary Figure 1: Cell proliferation remained unchanged in resistant cells.** Cells were stained with CFSE and the staining intensity was measured by flow cytometry. The percent of CFSE was normalized to the values observed at 24h after staining for each cell line. T-DM1 resistant cell lines proliferate at the same rate as the parental cell line.

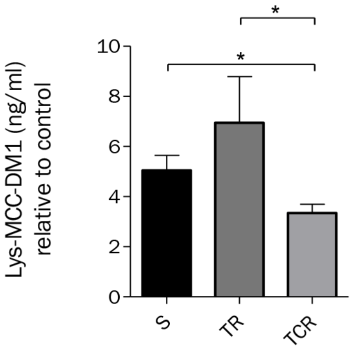


**Supplementary Figure 2: Lys-MCC-DM1 accumulation after exposure to T-DM1 is decreased in TCR cells compared to parental cells.** Lys-MCC-DM1 quantification by LC-MS/MS after 1 hour exposure to T-DM1 shows a decreased amount of the metabolite in TCR cells compared to TR and S cells (*: P<0,05).

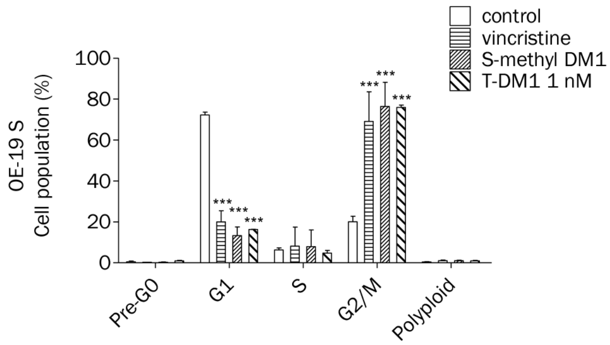


**Supplementary Figure 3: Cell cycle arrest is induced by T-DM1, vincristine and S-methyl DM1 in OE-19 parental cell line.** Cell cycle distribution determined by propidium iodide using flow cytometry after 24h exposure to 1 nM T-DM1, 1μM vincristine and 10 nM S-methyl DM1 shows a G2/M phase arrest in parental cells. Statistics analysis comparing cells exposed to the cytotoxic agents to control condition was performed by Two-way ANOVA followed by bonferroni posttest (***: P<0,001).

**A**

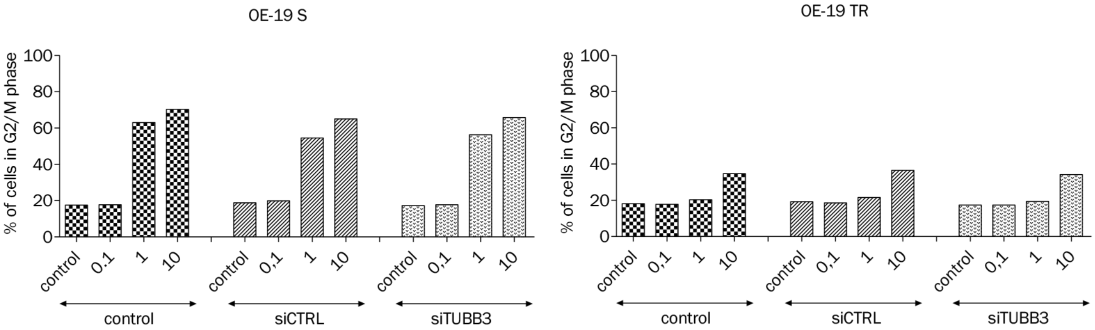


| | TUBB3 fold-change (siRNA/control) Arbitrary PCR units | |
|---|---|---|
| | OE-19 S | OE-19 TR |
| siCTRL | 1,2 | 0,9 |
| siTUBB3 | 0,3 | 0,2 |

**B**

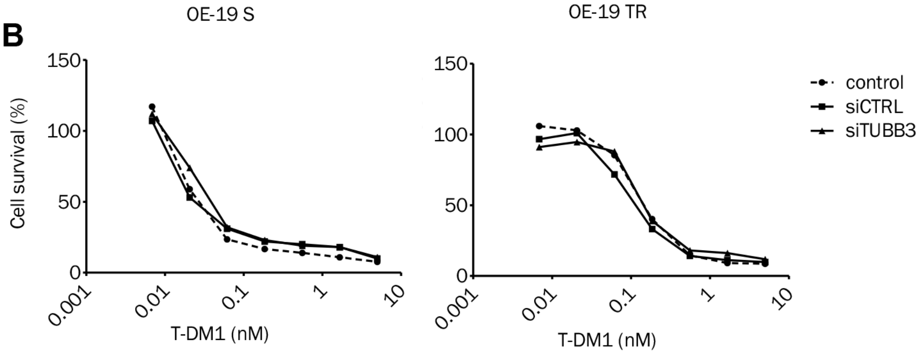


| | TUBB3 fold-change (siRNA/control) Arbitrary PCR units | |
|---|---|---|
| | OE-19 S | OE-19 TR |
| siCTRL | 1,4 | 0,8 |
| siTUBB3 | 0,1 | 0,1 |

**Supplementary Figure 4: Downregulation of βIII tubulin by siRNA did not affect T-DM1-induced cell cycle arrest or T-DM1-cytotoxicity in OE-19 S and OE-19 TR cell lines.** Cells were transfected with siRNA control (siCTRL) or siRNA targeting βIII tubulin (siTUBB3) 24h prior exposure to T-DM1. The fold-change expression of TUBB3 was evaluated by RT-qPCR and normalized using non-transfected cells (control) and is indicated for each experiment at 24h post-tranfection. **(A)** Cell cycle distribution was studied by propidium iodide using flow cytometry after 24h exposure to increasing concentrations of T-DM1. Downregulation of βIII tubulin did not affect cell cycle distribution in OE-19 S or OE-19 TR cells. **(B)** Cell survival was determined by and MTT assay after 6 days exposure to T-DM1. The sensitivity to T-DM1 was not modified in OE-19 S or OE-19 TR cells transfected with siTUBB3.

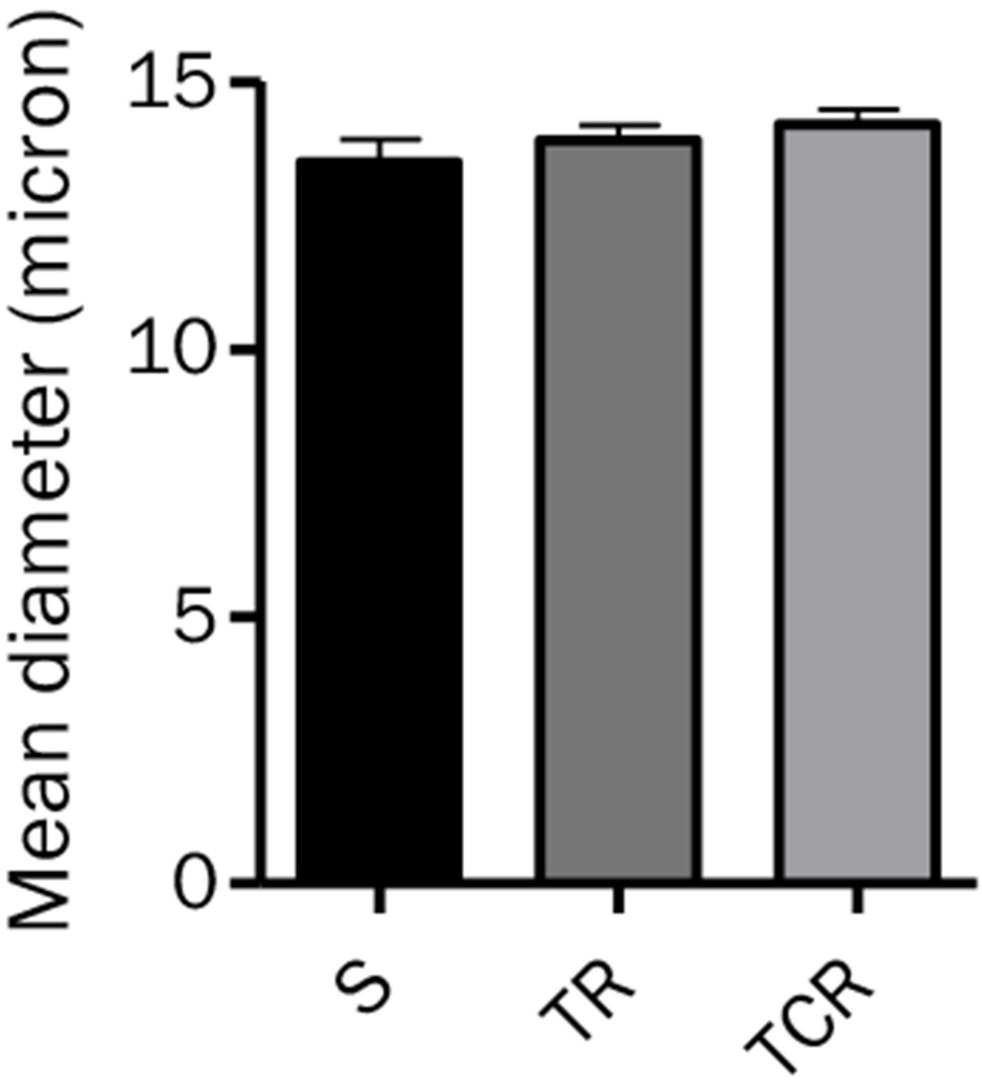


**Supplementary Figure 5: Cell size was not modified in resistant cells.** The mean diameter of cells in suspension measured by a Cellometer counter shows no modification of size in resistant cells compared to parental.

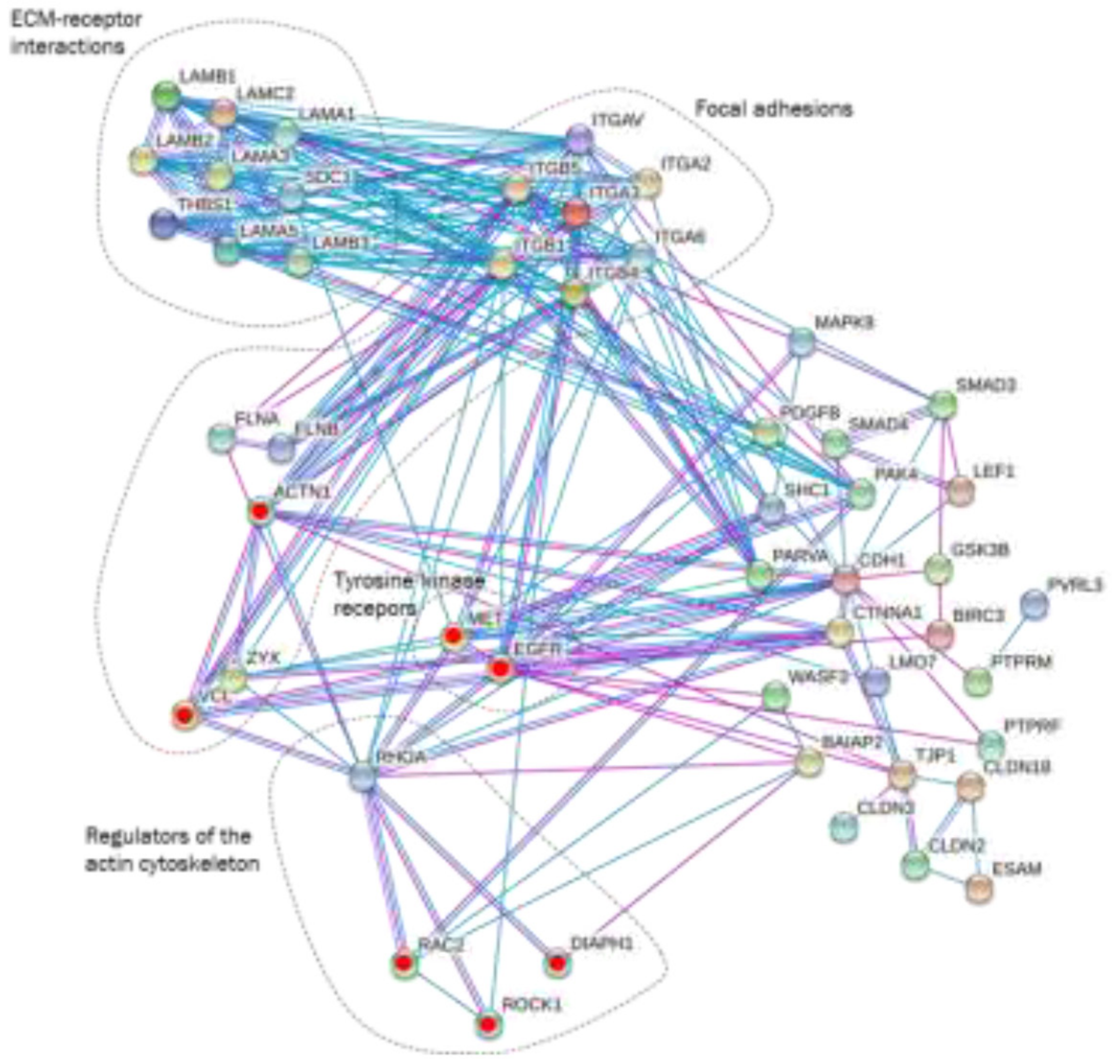


**Supplementary Figure 6: Network of proteins encoded by the genes deregulated in resistant cell lines.** The interactions between the genes listed in **Supplementary Table 1** were represented using STRING. Pink interactions are predicted from databases and blue interactions have been experimentally determined. The interactions were represented using a medium confidence score (0,400). The red dots indicate the molecules for which the gene expression was verified by RT-qPCR after the transcriptomic analysis, showed in Supplementary Table 2. RhoA did not appear to be deregulated but was represented since it is an important interactor of our molecules of interest.

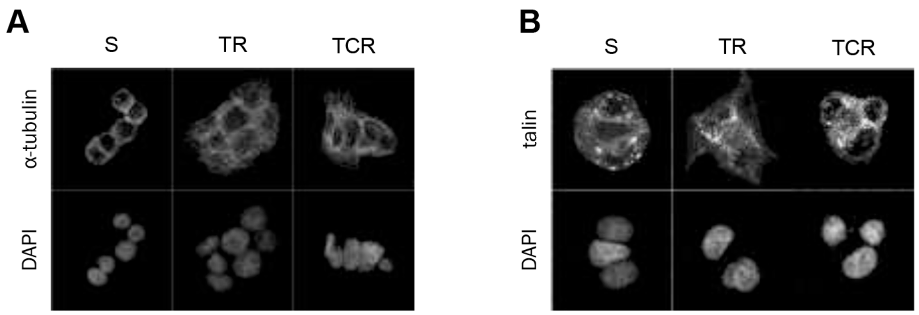


**Supplementary Figure 7: Single-channel images for tubulin and talin immunostaining. (A)** Immunostaining of α-tubulin and DAPI shows morphological changes in resistant cell lines compared to the parental cell line. **(B)** Immunostaining of talin and DAPI shows differences in size and amount of focal adhesions between parental and resistant cells.

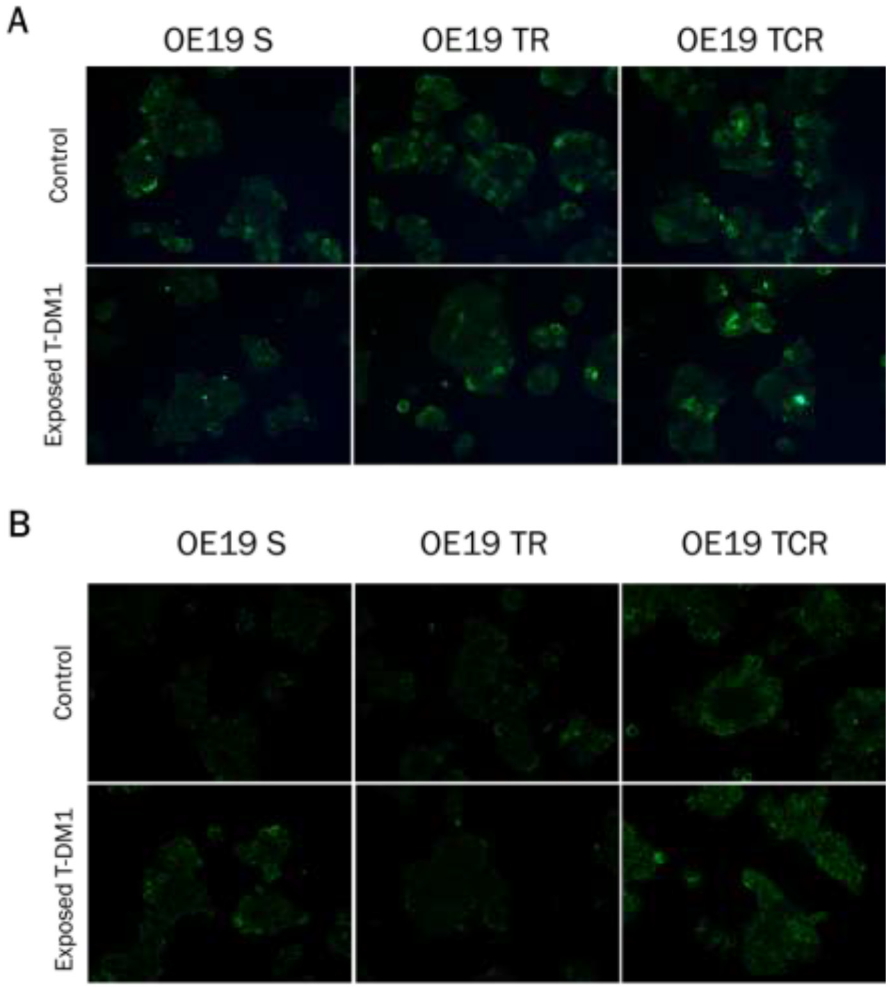


**Supplementary Figure 8: Immunostaining of actin and talin in parental and resistant cells.** **(A)** Immunostaining of β-actin does not suggest significant actin skeletal differences. **(B)** Immunostaining of talin was more pronounced in resistant cells at baseline but was not altered after exposure to T-DM1, whereas exposure of sensitive cells enhanced talin expression.

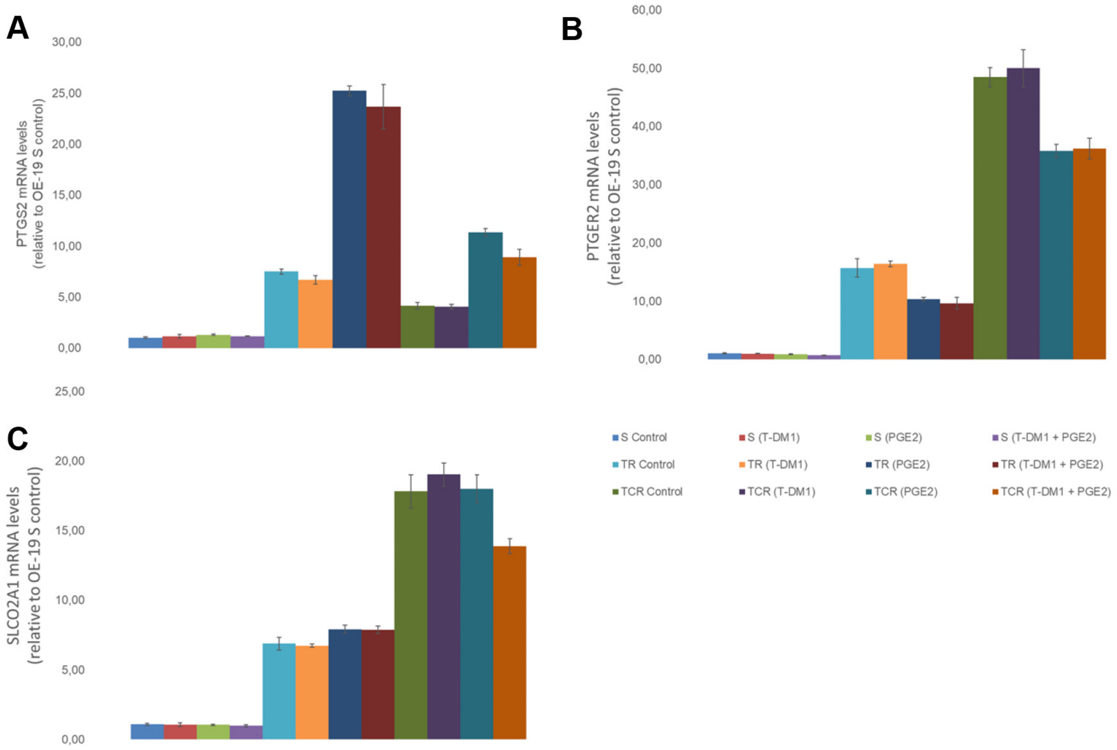


**Supplementary Figure 9: Expression levels of genes involved in COX-2 signaling pathway after exposure to Prostaglandin $E_2$.** Parental and resistant cell lines were exposed to 1nM T-DM1, 10μM $PGE_2$ or 1nM T-DM1 combined with 10μM $PGE_2$.mRNA levels were studied for PTGS2 (**A**), PGTER2 (**B**) and SLCO2A1 (**C**).

**Supplementary Table 1: Deregulated genes in OE-19 TR and OE-19 TCR cell lines compared to parental.** (**A**) Transcriptomic analysis of OE-19 S, TR and TCR cell lines shows several genes involved in cell adhesion that are deregulated in resistant cells compared to parental. **(B)** Expression fold change values of the genes of interest from the microarray and from RT-qPCR assays. The fold change was calculated as the level of expression in each resistant cell line over the one of the parental cell line ($^{**}$: P<0,01; $^{***}$: P>0,001).

**See Supplementary File 1**
