# Supplementary material for: Esophageal cancer cells resistant to T-DM1 display alterations in cell adhesion and the prostaglandin pathway
Source: Oncotarget. 2018 Apr 20;9(30):21141–55. doi: 10.18632/oncotarget.24975 (PMC5940380; doi:10.18632/oncotarget.24975)
Supplement: Supplementary file 2 [file oncotarget-09-21141-s002.docx]

Supplementary Table 1:

**A**

|  | Pathway | P-value | Genes |
| --- | --- | --- | --- |
| OE-19 TR | ECM-receptor interaction | 0,032 | *ITGA2, ITGA3 ITGA6, ITGAV, ITGB1, ITGB4, ITGB5, LAMA1, LAMA3, LAMA5, LAMB1, LAMB2, LAMB3, LAMC2, SDC1, THBS1* |
|  | Adherens junction | 0,033 | *SMAD3, SMAD4, WASF3, ACP1, ACTN1, CDH1, CTNNA1, EGFR, LEF1, MET, PVRL3, PTPRF, PAC2, TJP1, VCL* |
|  | Cell adhesion molecules | 0,041 | *CD276, CD58, CDH1, CLDN18, CLDN2, CLDN3, CNTNPA2, ESAM, GLG1, ALCAM, ITGA6, ITGB1, HLA-A, HLA-E, HLA-G, HLA-DRA, NRCAM, LOC647859, PVRL3, PTPRF, SDC1* |
|  | Focal adhesion | 0,058 | *SHC1, ACTN1, BIRC3 EGFR, FLNA, FLNB, GSK3B, ITGA2, ITGA3, ITGA6, ITGAV, ITGB1, ITBG4, ITGB5, LAMA1, LAMA3, LAMA5, LAMB1, LAMB2, LAMB3, LAMC2, MET, MAPK9, PAK4, PARVA, PDGFB, RAC2, THBS1, VCL, ZYX* |
| OE-19 TCR | Adherens junction | 0,002 | *BAIAP2, LMO7, SMAD3, SMAD4, WASF3, ACP1, ACTN1, CDH1, CTNNA1, EGFR, LEF1, MET, PVRL3, PTPRM, RAC2, TJP1* |

**B**

|  | | Fold change | | | |
| --- | --- | --- | --- | --- | --- |
|  |  | Microarray | | qPCR | |
|  |  | TR | TCR | TR | TCR |
| Tyrosine kinase receptors | *EGFR* | 1,4 | 1,3 | 1,8 ^(**)^ | 1,2 |
|  | *MET* | 1,5 | 1,4 | 1,8 ^(**)^ | 1,2 |
| Actin-interacting proteins | *ACTN1* | 1,6 | 1,7 | 2,1 ^(**)^ | 1,7 ^(**)^ |
|  | *VCL* | 1,7 |  | 1,9 ^(**)^ | 1,3 |
| Regulators of actin cytoskeleton | *ROCK1* |  | 1,4 | 2,0 ^(**)^ | 1,6 ^(**)^ |
|  | *RAC2* | 0,5 | 0,7 | 0,3 ^(***)^ | 0,5 ^(***)^ |
|  | *DIAPH1* |  |  | 1,9 ^(***)^ | 1,5 |

**Supplementary Table 1. Deregulated genes in OE-19 TR and OE-19 TCR cell lines compared to parental. A.** Transcriptomic analysis of OE-19 S, TR and TCR cell lines shows several genes involved in cell adhesion that are deregulated in resistant cells compared to parental. **B.** Expression fold change values of the genes of interest from the microarray and from RT-qPCR assays. The fold change was calculated as the level of expression in each resistant cell line over the one of the parental cell line (**: P<0,01; ***: P>0,001).
